# Combination of qualitative and quantitative methods for developing a new Health Related Quality of Life measure for patients with anogenital warts

**DOI:** 10.1186/1477-7525-3-24

**Published:** 2005-04-07

**Authors:** Xavier Badia, Jose Antonio Colombo, Nuria Lara, M Angels Llorens, Luis Olmos, Miguel Sainz de los Terreros, Jose Antonio Varela, Juan Jose Vilata

**Affiliations:** 1 Health Outcomes Research Europe Group, Avda. Diagonal, 618 1° C-D, 08021Barcelona, Spain; 2 Hospital Universitario Puerta del Mar, Cádiz, Spain; 3 Departamento Médico, 3M España, División Farmacéutica, Madrid, Spain; 4 Hospital Clínico, Madrid, Spain; 5 Unidad ITS, Gijón, Spain; 6 Hospital General Universitario, Valencia, Spain

## Abstract

**Background:**

Anogenital warts are the most easily recognized sign of genital Human Papilloma Virus infection. The objective was to develop a short, valid and reliable questionnaire to measure Health Related Quality of Life (HRQL) in patients with anogenital warts.

**Methods:**

First a literature review was performed to identify relevant papers describing the impact of anogenital warts in HRQL; second the main domains were identified by some experts in a focus group, and third in-depth-semi-structured interviews were conducted in patients with anogenital warts to identify the initial set of items. A qualitative reduction of the initial set of items was performed based on the mean scoring of the experts for the three scales: clarity, frequency and importance. The initial questionnaire was pilot tested in 135 patients. Rasch analysis was performed with the results of the questionnaire in order to refine the instrument. Spearman's correlation was calculated between the initial questionnaire and the reduced version. Additionally the measurement properties (validity and reliability) of the resulting final questionnaire were tested and compared using standard procedures (Cronbach's Alpha and item-total correlation).

**Results:**

the main domains identified as affected in patient's life were: sexual, colleagues and partner relationships. After a proper qualitative reduction the initial set of 134 items was reduced to 22. The questionnaire was pilot tested in 135 patients and two dimensions were identified after the multifactorial analysis: emotional dimension and sexual activity dimension. As a result of the Rasch analysis the questionnaire was reduced to 10 items. High correlation was found between the initial and the reduced version for the two dimensions. Cronbach's alpha values were acceptable (0.86).

**Conclusion:**

The initial 22 items questionnaire was reduced by Rasch analysis to a version of 10 items, with two dimensions: emotional and sexual. The results suggest the adequacy of the 10 items to evaluate HRQL of patients with anogenital warts in a valid and reliable way.

## Background

Any health problem may place significant restrictions on the normal development of physical, emotional and social aspects of a patient's life. Health Related Quality of Life (HRQL) is a multidimensional construct referring to patient's perceptions of the impact of disease and treatment on their physical, psychological, and social function and well-being [1]. The increasing interest in measures reflecting the personal viewpoint of patients' health has led to an extended demand for reliable and valid standardized questionnaires of Health-Related Quality of Life (HRQL) [2,3].

Although the use of HRQL questionnaires is more common in clinical research [4], their use in clinical practice can also help clinicians to obtain standardized information on the impact of the disease and its treatment on patients' HRQL; information that cannot be obtained using traditional measures, and that could be of a great use in clinical decision making.

Taking into account features like item content, scope and target population, instruments can be basically classified as generic or disease-specific [5]. Disease-specific questionnaires, those that only contain items specifically designed for a particular condition, are more likely to be relevant and sensitive to patients in areas that clinicians may wish to monitor [6,7].

Human Papilloma Virus (HPV) is one of the most common sexually transmitted infections. Its prevalence has increased many fold throughout the world, and it is estimated that a total of 1% of the sexually active population are infected. One study in Korea showed that among sexually active students, HPV prevalence was 38.8% in females and 10.6% in males [8]. Another study in Hungary showed an overall prevalence of HPV infection in women of 23% [9]. The number of sexual partners, frequency of sexual intercourse and the presence of genital warts in the partner, have been identified as factors that are related to HPV infection in women. In men only the number of sexual intercourses have shown to be related to the infection [10,11].

Genital warts (sometimes called condylomata acuminata or venereal warts) are the most easily recognized sign of genital HPV infection. Many people, however, have a genital HPV infection without genital warts. Genital warts are soft, moist, or flesh colored and appear in the genital area within weeks or months after infection. They sometimes appear in clusters that resemble cauliflower-like bumps, and are either raised or flat, small or large. Genital warts can show up in women on the vulva and cervix, and inside and surrounding the vagina and anus. In men, genital warts can appear on the scrotum or penis.

Quality of life of patients with genital warts is potentially affected [12]. Given the lack of a disease-specific instrument to measure HRQL in patients with anogenital warts, the objective of the study was to develop a short, valid and reliable questionnaire to measure quality of life in patients with anogenital warts.

## Methods

The process of questionnaire elaboration was divided in two phases:

### • Phase I: Generation of quality of life issues (domains) and items

A qualitative study was performed to assess the quality of life issues and to develop the items to be included in the anogenital warts disease-specific questionnaire.

The strategy was to first carry out a literature review to identify relevant papers describing the impact of anogenital warts in HRQL as well as to describe any questionnaires previously designed to measure HRQL in patients with anogenital warts. A semi-structured questionnaire was designed based on the results of the literature search. Secondly, a group of experts answered the semi-structured questionnaire in order to identify those domains of anogenital warts they believed would cause problems in patients' life. Afterwards a focus group was organized with the same ten experts. The moderator of the focus group was responsible for leading the discussion among experts in order to reach a final consensus about the dimensions to be considered in the final questionnaire.

### • Phase II

This was divided in the following steps:

1. Identification of the initial set of items through semi-structured interviews to a sample of patients.

2. Qualitative reduction of the initial set of items

3. Questionnaire design

4. Administration of the questionnaire to a sample of patients

5. Quantitative reduction of items, Rasch analysis.

#### 1. Identification of the initial set of items through semi-structured interview

With the aim of evaluating the impact of the symptoms of genital warts and its treatment in the quality of life, a group of patients were interviewed, using in-depth semi-structured-questionnaires. The interviewer made sure that the patient took into account all the dimensions previously identified by the experts and also tried to identify new dimensions that could arise during the interview. The interview focused on the impact of the disease on the physical, psychological and social functioning of the patient.

All semi-structured interviews were tape-recorded and transcribed to paper for the qualitative content analysis. A first qualitative analysis was based on the redundancy and repetition of expressions. As a result a first set of items was obtained.

#### 2. Qualitative reduction of the initial set of items

In a second meeting, the experts scored each one of the items. The criteria to include or exclude each one of the 134 items analyzed were based on the mean scoring given by the experts for the three scales: "clarity of wording", "frequency of occurrence", and "importance" among patients with anogenital warts, using a 1–5 Likert-type scale. Items were considered for final inclusion if clarity, frequency and importance were considered high, if clarity and importance were high together with low frequency, and also if frequency and importance were high and clarity low; in this latter situation the item was included but modified to enhance clarity. Low values were defined as those ones below percentile 50 of the distribution of the three scales, and high values where those ones above percentile 50. Based on the results of the scoring of each item a first item reduction was obtained.

#### 3. Questionnaire design and administration to a sample of patients

The pre- identified items were put together in a questionnaire format. The questionnaire was administered to a sample patients with anogenital warts.

#### 4. Quantitative reduction of the items

A multifactorial analysis was performed to define possible dimensions within the questionnaire. In order to refine the instrument a further quantitative reduction was performed with Rasch analysis (dichotomous logistic response model) [13], using the answers to the questionnaire from a group of patients with anogenital warts. The Rasch model constructs a line of measurement with the items placed hierarchically and provides fit statistics to indicate just how well different items describe the group of subjects responding to a questionnaire [14]. In this study a Infit and Outfit value below 1.3 was accepted. No definitive rules exist regarding what is considered acceptable and unacceptable fit, but values greater than 1.3, in a sample smaller than 500, are usually diagnosed as potential misfits to Rasch model condition [15].

A Spearman's correlation was obtained between the original questionnaire and the final reduced version. Additionally the measurement properties (validity and reliability) of the resulting final questionnaire were tested and compared using standard procedures (Cronbach's Alpha and item-total correlation).

## Results and Discussion

This study attempts to develop a disease-specific quality-of-life questionnaire for adults with anogenital warts. The fact that is a disease specific one allows the comparison between groups of patients within the same disease.

### Phase I: Generation of quality of life domains and items

Pubmed data base was searched [16]. No specific instrument to measure HRQL in patients with anogenital warts was found after the literature review. Only a few studies were found that had considered which dimensions of HRQL were affected in patients with anogenital warts. Based on the results of the literature research a semi-structured questionnaire was elaborated.

Three dermatologists and one gynaecologist answered the semi-structured questionnaire, as well as two experts in the elaboration of HRQL instruments. Given that anogenital warts are mainly seen by dermatologists and gynaecologists in Spain, these two specializations were chosen in order to get the widest perspective of the population affected with anogenital warts and the aspects that impact their quality of life. Three aspects were identified as having impact on HRQL of patients with anogenital warts: sexual relationships, relationships with colleagues, and within the couple relationship. The aspects that worry the patient mentioned by the participants were: contagion, cancer, guilt, anticonceptive methods, pregnancy, infidelity and low performance. In men the most affected aspects were sexual relationships (continuity of sexual relationships, fear to contagion and use of condom). In women the most affected aspects were maternity (fear to pregnancy and possibility to contagion for the baby), fear to cancer, sexual relationships and within couple relationship.

Finally all participants agreed that the aspects that most impact HRQL of patients with anogenital warts are: couple relationship, guilt, and fear of contagion. It is worth pointing out that at this initial state none of the experts mentioned the patient's worry about the effectiveness of the treatment, while all the aspects were more related to patient's relationships.

With all the information gathered in phase I a guideline for the semi-structured interview of patients was elaborated.

### Phase II

#### Identification of the initial set of items

A total of 20 in-depth semi-structured interviews of patients with anogenital warts were obtained between June and July 2003. The sample of 20 patients is considered to be more than enough in qualitative research at the stage of identifying dimensions [17]. The participants were 11 men and 9 women, mean age 34.2, ranging from 19 to 53. Mean time since diagnosis was 25.3 months, ranging from 15 years (1 patient) to 1 month (2 patients). The treatment received by the patients was: criotherapy in 9 cases, inmunomodulators in 2 cases, and no treatment in 9 cases. The interviews were conducted by two experienced psychologists.

The focus group technique could have also been used in this step. The advantages of focus group is that they do not discriminate against patients who cannot read or write and they can encounter participation from people reluctant to be interviewed. [18]. In this case a semi-structured interviewed was chosen because the initial domains were already identified, therefore the semi-structured interviewed was good to provide information on concrete issues allowing open answers.

The dimensions identified were: physical, psychological, social, sexual, everyday life activities, symptoms, perception of the disease, perception of health and perception of treatment. After the qualitative analysis, based on the redundancy and repetition of expressions, a first set of 134 items was obtained.

The selection of items was based on patient's opinion and health professional's views. The aim of this methodological approach is to ensure that the content and validity (specificity and sensibility) of the tool under construction is appropriate for the target population. In other words, whether the questionnaire deals with relevant questions on disease and treatment and does not omit important patient issues [19]. As we see in this study, treatment issues were not initially mentioned by experts while the issue was raised by the patients later on.

Regarding the patient issues the results do not differ from the ones found in the literature. In a survey by the American Social Health Association among people with human papillomavirus infection, more than three-quarters of respondents reported feelings of depression and anger, and two-thirds feelings of shame. Sexual enjoyment and sexual activity were also negatively affected by genital warts [20]. Other studies have shown a negative impact in the social life of the patient [12].

It has been demonstrated that the help seeking behaviour in patients with sexually transmitted diseases is determined by the shame and stigma that they feel [21-23]. This fact might limit the results of the study because those patients who did not seek for health care due to shame and stigma were not able to participate in the study.

#### Qualitative reduction of the initial set of items

The criteria to include or exclude each one of the 134 items analyzed were based on the mean scoring given by the experts for the three scales: "clarity of wording", "frequency of occurrence", and "importance". A total of 49 items were included.

A further qualitative review of the pre-selected items based on prior experience with other similar instruments concluded in the initial 22 -item questionnaire, which was called CECA 22 (Cuestionario Específico en Condilomas Acuminados, in Spanish) (Table 1). The questions referred to the previous 7 days before the visit, and each question allowed an answer in a 5 options Likert scale (always, almost always, sometimes, rarely, never) with the exception of items 3, 5, and 21 that also allowed the option of "not applicable". The higher the score the better the quality of life.

**Table 1:** Results of the administration of the 22 item questionnaire to a sample of patients. The items of the final 10-item questionnaire are highlighted in bold

N° of Item	Item	n	Range		Mean scoring (SD)
			Min	Max	
1	The discomfort that they cause me affects my daily activites.	135	1	5	3.5 (1.2)
2	I worry too much about my personal hygiene	135	1	5	1.7 (0.9)
3	**I am afraid that the lesions won't disappear**	135	1	5	2.4 (1.4)
4	**I am anxious to know whether I am going to recover from the infection for good**	134	1	5	1.7 (1.1)
5	**I worry about whether the warts will get worse or if there will be some complications**	135	1	5	2.1 (1.3)
6	I have feelings of guilt	135	1	5	3.3 (1.4)
7	I am anxious to know who infected me and when	135	1	5	2.2 (1.4)
8	I worry that I might infect other people	135	1	5	1.4 (0.8)
9	**My state of mind is upset (anxiety, depression, sadness, uneasiness...)**	135	1	5	2.9 (1.2)
10	**I feel more insecure**	135	1	5	2.9 (1.3)
11	**Knowing that I have the illness affects me in my daily life**	135	1	5	3.3 (1.3)
12	I worry about whether I might have problems in having children in the future	135	1	5	3.7 (1.5)
13	I worry about the lack of knowledge concerning my illness and its treatment	135	1	5	2.7 (1.5)
14	I worry about people finding out about my illness (at work, at leisure centres, etc.)	135	1	5	2.4 (1.6)
15	My personal relationships have been affected (partner, friendship,	134	1	5	3.1 (1.4)
16	**My sexual drive has decreased**	135	1	5	3.4 (1.4)
17	**I feel worried during the act**	135	1	5	2.8 (1.4)
18	**I avoid sexual relations**	134	1	5	3.3 (1.4)
19	**My sexual relations have decreased in quality and/or frequency**	135	1	5	3.0 (1.4)
20	I worry about having to use barrier methods (condom, diaphragm) to have sexual relation	135	1	5	3.5 (1.6)
21	The discomfort produced by treatment affects my daily life	135	1	5	3.7 (1.3)
22	I worry about having to follow a treatment for a long time	135	1	5	2.4 (1.4)

#### Administration of the questionnaire to a sample of patients

The 22 item questionnaire was pilot tested in a sample of 135 patients diagnosed of anogenital warts from different centers throughout Spain. Mean age (SD) was 31.1 (7.3), 51% of them were women. A total of 76.3% of the patients were heterosexual and 17.8% homosexual. In 32.1% of the patients the anogenital warts were not visible at the moment of the visit. In 6% the extension of the wart was large (>200 mm^2^), in 29.6% was medium (50–200 mm^2^) and small in 35.1% (< 50 mm^2^). The anogenital warts were mostly located in the penis in men and vulva in women. The diversity of the patients' disease situation enhanced the possibility of getting as many broad results as possible.

The questionnaire was completely fulfilled by the 132 patients (response rate 132/135). The results of the administration are shown in table 1. Sexual activities and everyday life activities, as well the worry for having children were the dimensions that obtained higher scoring, meaning that they were the main worry for the patient.

#### Quantitative reduction of the items

The multifactorial analysis identified two possible dimensions in the preliminary CECA questionnaire. The first one (emotional dimension) was integrated by the first 15 items, and the second (sexual activity dimension) was integrated by the 7 remaining items. Afterwards the Rasch analysis was performed for each one of the dimensions. Rasch model enumerates the items in relation to gravity and selects the items according to: adjustment to the model, redundancy, discriminant validity, and content. As a result of it, the first dimension initially formed by 15 items was reduced to a total of 6 items including: item 3, 4, 5, 9, 10 and 11. There was evidence of the overlapping of item 9 and 10 but the decision of excluding or keeping any of them was totally qualitative. The final decision was to keep the six items. The second dimension (7 items) was reduced to 4 items, including : item 16, 17, 18 and 19. Therefore the final version of the questionnaire had a total of 10 items (Table 1). Global scoring range was 10 to 50, going from 6 to 30 in the emotional dimension and from 4 to 20 in sexual activity dimension. All dimensions were standardized for a scoring between 0 (worst HRQL) and 100 (the best HRQL) in order to facilitate the interpretation and comprehension.

Afterwards a global final analysis was performed to evaluate the adequacy of the total of the selected items in the final reduced version. The correlation between the reduced version and the two dimensions (emotional and sexual activity) in the 22 item questionnaire was high (Table 2). The results of the item-total correlation and the reliability (Cronbach's alpha) for the reduced version as well as for the initial 22-items one are shown in table 3. Item total correlation is the correlation coefficient between the score on an individual item and the total score for the whole set of items of which the individual item is a part. It provides a clue of the internal consistency, the higher the value the higher the consistency (minimum recommended value 0.3).

**Table 2:** Correlation between the reduced version and the two dimensions (emotional and sexual activity) in the 22 item questionnaire

	CECA10	CECA6 (emotional)	CECA4 (sexual activity)
CECA6 (emotional)	r = 0.851* n = 133		
CECA4 (sexual activity)	r = 0.830* n = 133	r = 0.433* n = 133	
CECA22	r = 0.928* n = 133	r = 0.845* n = 132	r = 0.719* n = 132

* p < 0.01

CECA 22: initial 22-item questionnaire

CECA 10: 10-item questionnaire

CECA 6: emotional dimension in the 10-items questionnaire

CECA 4 sexual activity dimension in the 4-item questionnaire

**Table 3:** Results of the item-total correlation and the reliability (Cronbach's alpha) for the reduced version as well as for the initial 22-item

		CECA22*	CECA10	CECA6	CECA4
N° of items		22	10	6	4

Scoring distribution					

Valid observations		132	133	134	134
Mean		44.9	44.6	38.8	53.4
SD		17.5	22.3	23.9	29.3
Percentile 50		43.7	40	37.5	53.1
% scoring 0		0	0	5	3 (2.2%)
% scoring 100		0	1	2	8 (5.9%)
Correlation item-global scoring		0.21–0.72	0.53–0.73	0.65–0.79	0.78–0.89
Reliability (Cronbach's alpha)		0.87	0.86	0.84	0.86
Results of Rasch analysis	Person separation	2.28	2.21	1.87	1.79
	Person reliability	0.84	0.83	0.78	0.76

CECA 22: initial 22-item questionnaire

CECA 10: 10-item questionnaire

CECA 6: emotional dimension in the 10-items questionnaire

CECA 4: sexual activity dimension in the 4-item questionnaire

The mean (SD) scoring for CECA-10, in a 0 to 100 scale, was 44,6 (Table 4). The CECA-6 factor showed a mean (SD) of 38,8 (23.9) and the factor CECA-4 53.4 (29.3). Patients showed worse quality of life (more impact) in the emotional dimension.

**Table 4:** Global scoring of the CECA 10 questionnaire and for each one of the dimensions

	Mean	Median	Standard deviation	Minimum	Maximum	N Valid
Global scoring CECA-10 0-worse HRQL 100 better HRQL	44.61	40.00	22.33	2.50	100.00	133
Global scoring CECA-6	38.84	37.50	23.93	.00	100.00	134
Global scoring CECA -4	53.6	56.25	29.8	.00	100.00	134

CECA-6: emotional dimension

CECA-4: sexual activity dimension

Even though the final questionnaire was initially designed to be equally valid for both genders, it would be interesting in future research to develop gender specific questionnaires given the characteristics of the disease.

## Conclusion

The original CECA questionnaire composed by 22 items was reduced by Rasch analysis to a version of 10 items. Two dimensions were identified in the final questionnaire: emotional and sexual activity. The results suggest the adequacy of the 10 items to evaluate in a valid and reliable way the HRQL of patients with anogenital warts.
